# Development and validation of a prediction model of hospital mortality for patients with cardiac arrest survived 24 hours after cardiopulmonary resuscitation

**DOI:** 10.3389/fcvm.2025.1510710

**Published:** 2025-01-27

**Authors:** Renwei Zhang, Zhenxing Liu, Yumin Liu, Li Peng

**Affiliations:** ^1^ Department of Neurology, Zhongnan Hospital of Wuhan University, Wuhan, China; ^2^ Department of Neurology, Yiling Hospital of Yichang, Yichang, China; ^3^ Department of Cardiology, Zhongnan Hospital of Wuhan University, Wuhan, China

**Keywords:** hospital mortality, nomogram, cardiac arrest, LASSO, cardiopulmonary resuscitation

## Abstract

**Objective:**

Research on predictive models for hospital mortality in patients who have survived 24 h following cardiopulmonary resuscitation (CPR) is limited. We aim to explore the factors associated with hospital mortality in these patients and develop a predictive model to aid clinical decision-making and enhance the survival rates of patients post-resuscitation.

**Methods:**

We sourced the data from a retrospective study within the Dryad dataset, dividing patients who suffered cardiac arrest following CPR into a training set and a validation set at a 7:3 ratio. We identified variables linked to hospital mortality in the training set using Least Absolute Shrinkage and Selection Operator (LASSO) regression, as well as univariate and multivariate logistic analyses. Utilizing these variables, we developed a prognostic nomogram for predicting mortality post-CPR. Calibration curves, the area under receiver operating curves (ROC), decision curve analysis (DCA), and clinical impact curve were used to assess the discriminability, accuracy, and clinical utility of the nomogram.

**Results:**

The study population comprised 374 patients, with 262 allocated to the training group and 112 to the validation group. Of these, 213 patients were dead in the hospital. Multivariate logistic analysis revealed age (OR 1.05, 95% CI: 1.03–1.08), witnessed arrest (OR 0.28, 95% CI: 0.11–0.73), time to return of spontaneous circulation (ROSC) (OR 1.05, 95% CI: 1.02–1.08), non-shockable rhythm (OR 3.41, 95% CI: 1.61–7.18), alkaline phosphatase (OR 1.01, 95% CI: 1–1.01), and sequential organ failure assessment (SOFA) (OR 1.27, 95% CI: 1.15–1.4) were independent risk factors for hospital mortality for patients who survived 24 h after CPR. ROC of the nomogram showed the AUC in the training and validation group was 0.827 and 0.817, respectively. Calibration curves, DCA, and clinical impact curve demonstrated the nomogram with good accuracy and clinical utility.

**Conclusion:**

Our prediction model had accurate predictive value for hospital mortality in patients who survived 24 h after CPR, which will be beneficial for assisting in identifying high-risk patients and intervention. Further confirmation of the model's accuracy required external validation data.

## Introduction

In the United States, 420,000 people suffer from out-of-hospital cardiac arrest each year, and the survival rate is only about 6% (1, 2). Early assessment in patients with cardiac arrest can improve their prognosis and reduce the mortality rate (3). Cardiopulmonary resuscitation (CPR) is the only effective treatment for cardiac arrest (4–6). In recent years, remarkable progress has been made in the field of CPR, such as the optimization of compression techniques, the popular application of automated external defibrillators (AEDs), and comprehensive treatment strategies after CPR. Every innovation and improvement may have a significant impact on the survival rate of patients. The widespread dissemination of education and training has enabled more members of the public to master this life-saving skill, thereby providing timely assistance in emergencies. Despite recent progress in resuscitation medicine and critical care medicine, the mortality rate after cardiac arrest remains high, especially in the case of out-of-hospital cardiac arrest (7). Accurate prognosis assessment is an important method for improving treatment efficiency, enhancing outcomes, preserving patient dignity, and reducing the burden of cardiac arrest.

Several studies have developed predictive models for hospital mortality following CPR, yet these models possess certain limitations, including their application primarily to pediatric patients or those experiencing out-of-hospital cardiac arrest, suboptimal prediction accuracy, and the omission of clinical laboratory variables (8–11). Therefore, we aimed to investigate the variables associated with hospital mortality for patients who survived 24 h after CPR, and then construct a predictive model that can guide clinical treatment decisions and improve resuscitation survival.

## Methods and materials

### Data resource

The data resource in this study are available from the Dryad Digital Repository (https://datadryad.org/stash/dataset/doi:10.5061/dryad.qv6fp83), which is an open data publishing platform devoted to the open availability and reuse of all research data.

### Study population

This retrospective study was conducted by Iesu et al. (12) in the Intensive Care Unit of Erasme Hospital in Brussels (Belgium). 435 patients with coma (Glasgow Coma Scale, GCS < 9) after cardiac arrest were recruited in the study from January 2007 to December 2015, 61 patients with missing clinical data or death less than 24 h after admission were excluded, and finally 374 patients were included in our analysis. Patients were treated with standard post-resuscitation management broadly described elsewhere (12, 13).

### Data collection

We collected these data for analysis in our study: (1) demographics: age, sex and weight; (2) comorbidities: chronic heart failure, hypertension, coronary artery disease, diabetes, chronic obstructive pulmonary disease (COPD)/asthma, neurological disease, chronic renal failure, acute kidney injury (AKI), liver cirrhosis, HIV; (3) arrest characteristics: witnessed, time to return of spontaneous circulation (ROSC), adrenaline, out of hospital, non-shockable rhythm, ICU mortality, hospital mortality, by stander CPR; (4) Severity of disease: Acute Physiology and Chronic Health Evaluation (APACHE) II score, Sequential Organ Failure Assessment (SOFA) score; (5) laboratory test: lactate, central venous oxygen saturation or mixed venous oxygen saturation (ScvO2/SvO2), aspartate aminotransferase (AST), alanine aminotransferase (ALT), lactate dehydrogenase (LDH), alkaline phosphatase, γ-glutamine transferase (GGT), total bilirubin, activated partial thromboplastin time (APTT), prothrombin time (PT), international normalized ratio (INR), platelets, proteins, glucose, pH, PaCO2, PaO2, mean arterial pressure (MAP), creatine, C-reactive protein (CRP); (6) treatment during hospital: targeted temperature management (TTM), intra-aortic balloon counter pulsation (IABP), extracorporeal membrane oxygenation (ECMO), mechanical ventilation, continuous renal replacement therapy (CRRT).

### Statistical analysis

SPSS 27 and R 4.1.3 software were used in our analysis. Continuous data was presented as X ± S and a comparison between two groups was used *t*-test. Nonparametric variables were supplied as median [interquartile range (IQR)] and comparison between groups using the Mann–Whitney *U*-test. When appropriate, categorical variables were assessed using Fisher's exact or the *χ*^2^ test and given as numbers (percentage). The dataset is partitioned into a training set and a validation set at a ratio of 7:3. Within the training set, variable selection is initially conducted using LASSO (Least Absolute Shrinkage and Selection Operator) regression, which is a regression analysis method that performs both variable selection and regularization to enhance the prediction accuracy and interpretability of the statistical model by shrinking the coefficients of less important predictors to exactly zero. Following this, univariate logistic regression is applied to further screen the variables. Variables with univariate *P*-values less than 0.05 are included in the multivariate logistic regression analysis to identify independent risk factors associated with the prognosis. Finally, a nomogram prediction model is constructed based on the variables selected through this rigorous process. The prediction model was evaluated by ROC, calibration curve, DCA, and clinical impact curve. The difference with *P* < 0.05 was statistically significant.

## Results

### Baseline characteristics of the training and test group

A total of 435 patients were enrolled in the study. However, 61 patients were excluded due to missing clinical data or death occurring within 24 h of admission. Finally 374 patients were included in our analysis, with 262 patients assigned to the training group and 112 patients to the validation group. Of these, 213 patients succumbed to their condition in the hospital. The baseline characteristics for the training and validation groups were presented in Table 1.

**Table 1:** Baseline characteristics of the training and validation group.

Variables	All *N* = 374	The training group *N* = 262	The validaiton group *N* = 112	*P* value
Age, years	62.00 [51.25; 74.00]	62.00 [52.00; 74.75]	62.50 [50.75; 72.25]	0.425
Weight, kg	77.00 [67.25; 85.00]	76.50 [67.25; 85.00]	79.50 [67.75; 85.00]	0.379
Gender, *n* (%)				0.733
Female	104 (27.81%)	71 (27.10%)	33 (29.46%)	
Male	270 (72.19%)	191 (72.90%)	79 (70.54%)	
Hypertension, *n* (%)				0.104
No	215 (57.49%)	143 (54.58%)	72 (64.29%)	
Yes	159 (42.51%)	119 (45.42%)	40 (35.71%)	
Diabetes, *n* (%)				0.076
No	283 (75.67%)	191 (72.90%)	92 (82.14%)	
Yes	91 (24.33%)	71 (27.10%)	20 (17.86%)	
Coronary artery disease, *n* (%)				0.329
No	228 (60.96%)	155 (59.16%)	73 (65.18%)	
Yes	146 (39.04%)	107 (40.84%)	39 (34.82%)	
COPD/Asthma, *n* (%)				1.000
No	311 (83.16%)	218 (83.21%)	93 (83.04%)	
Yes	63 (16.84%)	44 (16.79%)	19 (16.96%)	
Chronic heart failure, *n* (%)				1.000
No	296 (79.14%)	207 (79.01%)	89 (79.46%)	
Yes	78 (20.86%)	55 (20.99%)	23 (20.54%)	
Previous neurologic disease, *n* (%)				0.238
No	320 (85.56%)	220 (83.97%)	100 (89.29%)	
Yes	54 (14.44%)	42 (16.03%)	12 (10.71%)	
Chronic renal failure, *n* (%)				0.273
No	311 (83.16%)	222 (84.73%)	89 (79.46%)	
Yes	63 (16.84%)	40 (15.27%)	23 (20.54%)	
Liver cirrhosis, *n* (%)				0.749
No	357 (95.45%)	249 (95.04%)	108 (96.43%)	
Yes	17 (4.55%)	13 (4.96%)	4 (3.57%)	
Corticoids, *n* (%)				0.797
No	289 (77.27%)	201 (76.72%)	88 (78.57%)	
Yes	85 (22.73%)	61 (23.28%)	24 (21.43%)	
Chronic anticoagulation, *n* (%)				0.544
No	309 (82.62%)	219 (83.59%)	90 (80.36%)	
Yes	65 (17.38%)	43 (16.41%)	22 (19.64%)	
Witnessed arrest, *n* (%)				0.829
No	54 (14.44%)	39 (14.89%)	15 (13.39%)	
Yes	320 (85.56%)	223 (85.11%)	97 (86.61%)	
Bystander CPR, *n* (%)				0.556
No	120 (32.09%)	87 (33.21%)	33 (29.46%)	
Yes	254 (67.91%)	175 (66.79%)	79 (70.54%)	
Time to ROSC, min	15.00 [7.00; 25.00]	15.00 [7.00; 25.00]	14.50 [7.00; 24.00]	0.495
Non cardiac etiology, *n* (%)				0.762
No	221 (59.09%)	153 (58.40%)	68 (60.71%)	
Yes	153 (40.91%)	109 (41.60%)	44 (39.29%)	
Non-shockable rhythm, *n* (%)				0.125
No	153 (40.91%)	100 (38.17%)	53 (47.32%)	
Yes	221 (59.09%)	162 (61.83%)	59 (52.68%)	
Epinephrine, mg	3.00 [2.00; 5.00]	3.00 [1.25; 5.00]	3.00 [2.00; 5.00]	0.724
Out of hospital, *n* (%)				0.046
No	166 (44.39%)	107 (40.84%)	59 (52.68%)	
Yes	208 (55.61%)	155 (59.16%)	53 (47.32%)	
TTM, *n* (%)				0.978
No	42 (11.23%)	30 (11.45%)	12 (10.71%)	
Yes	332 (88.77%)	232 (88.55%)	100 (89.29%)	
Hospital death, *n* (%)				0.328
No	161 (43.05%)	108 (41.22%)	53 (47.32%)	
Yes	213 (56.95%)	154 (58.78%)	59 (52.68%)	
IABP, *n* (%)				0.752
No	350 (93.58%)	244 (93.13%)	106 (94.64%)	
Yes	24 (6.42%)	18 (6.87%)	6 (5.36%)	
ECMO, *n* (%)				0.592
No	327 (87.43%)	227 (86.64%)	100 (89.29%)	
Yes	47 (12.57%)	35 (13.36%)	12 (10.71%)	
Shock, *n* (%)				0.753
No	174 (46.52%)	120 (45.80%)	54 (48.21%)	
Yes	200 (53.48%)	142 (54.20%)	58 (51.79%)	
ICU Vasopressor therapy, *n* (%)				0.645
No	91 (24.33%)	66 (25.19%)	25 (22.32%)	
Yes	283 (75.67%)	196 (74.81%)	87 (77.68%)	
ICU dobutamine, *n* (%)				0.702
No	173 (46.26%)	119 (45.42%)	54 (48.21%)	
Yes	201 (53.74%)	143 (54.58%)	58 (51.79%)	
CRRT, *n* (%)				0.815
No	313 (83.69%)	218 (83.21%)	95 (84.82%)	
Yes	61 (16.31%)	44 (16.79%)	17 (15.18%)	
Amiodarone, *n* (%)				0.910
No	187 (50.00%)	130 (49.62%)	57 (50.89%)	
Yes	187 (50.00%)	132 (50.38%)	55 (49.11%)	
Beta lactams, *n* (%)				0.383
No	216 (57.75%)	147 (56.11%)	69 (61.61%)	
Yes	158 (42.25%)	115 (43.89%)	43 (38.39%)	
AKI, *n* (%)				0.876
No	153 (40.91%)	106 (40.46%)	47 (41.96%)	
Yes	221 (59.09%)	156 (59.54%)	65 (58.04%)	
Lowest ScvO2, %	62.00 [56.10; 66.10]	62.00 [56.00; 66.47]	62.00 [56.95; 66.00]	0.953
Minimum platelets, /mm^3^	132.50 [79.25; 187.00]	132.50 [72.50; 183.25]	132.50 [88.75; 193.50]	0.356
Lactate, mEq/L	5.10 [4.10; 7.70]	5.15 [4.20; 7.60]	4.70 [3.88; 7.80]	0.188
CRP, mg/dl	40.00 [14.25; 83.50]	40.00 [16.50; 80.00]	35.00 [9.00; 90.00]	0.330
Creatinine, mg/dl	1.20 [0.90; 1.60]	1.20 [0.90; 1.60]	1.20 [0.90; 1.50]	0.386
ScvO2/SvO2 on admission, %	68.85 [63.70; 74.40]	68.75 [63.28; 74.30]	69.00 [64.60; 74.75]	0.299
AST, IU/L	95.00 [47.00; 192.50]	100.50 [51.00; 208.25]	85.50 [40.00; 151.25]	0.063
ALT, IU/L	68.00 [32.00; 152.75]	72.00 [33.00; 166.00]	58.50 [30.00; 113.50]	0.044
LDH, IU/L	335.50 [240.00; 488.00]	343.50 [245.25; 486.75]	329.50 [233.75; 489.50]	0.435
Alkaline phosphatase, IU/L	77.00 [58.00; 106.00]	74.50 [59.00; 101.75]	81.50 [57.00; 116.50]	0.519
GGT, IU/L	69.00 [43.00; 102.00]	69.00 [43.00; 101.49]	72.50 [42.00; 104.00]	0.822
Total bilirubin, mg/dl	0.51 [0.33; 0.86]	0.51 [0.33; 0.87]	0.51 [0.35; 0.85]	0.733
APTT, sec	32.30 [27.25; 44.10]	33.25 [27.40; 44.72]	31.50 [27.12; 42.25]	0.284
PT, %	65.00 [47.00; 79.00]	62.00 [46.00; 77.75]	69.00 [53.00; 81.25]	0.014
INR	1.26 [1.12; 1.54]	1.30 [1.12; 1.61]	1.21 [1.12; 1.42]	0.027
Platelets on admission, /mm^3^	201.00 [138.25; 266.25]	201.00 [138.25; 250.75]	199.00 [138.75; 282.00]	0.506
Protein, mg/dl	5.70 [5.00; 6.40]	5.70 [5.10; 6.50]	5.70 [5.00; 6.23]	0.444
Glucose, mg/dl	199.50 [155.00; 289.25]	206.50 [152.25; 293.75]	190.50 [159.75; 278.25]	0.273
pH	7.30 [7.21; 7.38]	7.29 [7.20; 7.38]	7.30 [7.22; 7.39]	0.445
PaO2, mmHg	111.00 [85.00; 178.00]	119.00 [87.00; 183.00]	104.50 [84.75; 152.00]	0.076
PaCO2, mmHg	37.00 [33.00; 44.00]	37.00 [32.00; 44.00]	39.00 [34.00; 44.00]	0.176
MAP, mmHg	86.00 [75.25; 103.00]	86.00 [75.00; 103.00]	89.00 [76.75; 105.25]	0.475
APACHE II	25.00 [20.00; 29.00]	25.00 [20.00; 29.00]	24.00 [20.00; 30.00]	0.855
SOFA	11.00 [9.00; 14.00]	11.00 [9.00; 14.00]	10.50 [8.00; 14.00]	0.117

COPD, chronic obstructive pulmonary disease; ROSC, return of spontaneous circulation; APACHE II, acute physiology and chronic health evaluation score; SOFA, sequential organ failure assessment score; ScvO2/SvO2, central venous oxygen saturation or mixed venous oxygen saturation; AST, aspartate aminotransferase; ALT, alanine aminotransferase; LDH, lactate dehydrogenase; GGT, γ-glutamine transferase; APTT, activated partial thromboplastin time; PT, prothrombin time; INR, international normalized ratio; MAP, mean arterial pressure; CRP, C-reactive protein; TTM, targeted temperature management; IABP, intra-aortic balloon counter pulsation; ECMO, extracorporeal membrane oxygenation; CRRT, mechanical ventilation, continuous renal replacement therapy; AKI, acute kidney injury.

### Variables associated with hospital mortality in the training group

**Figure 1:**
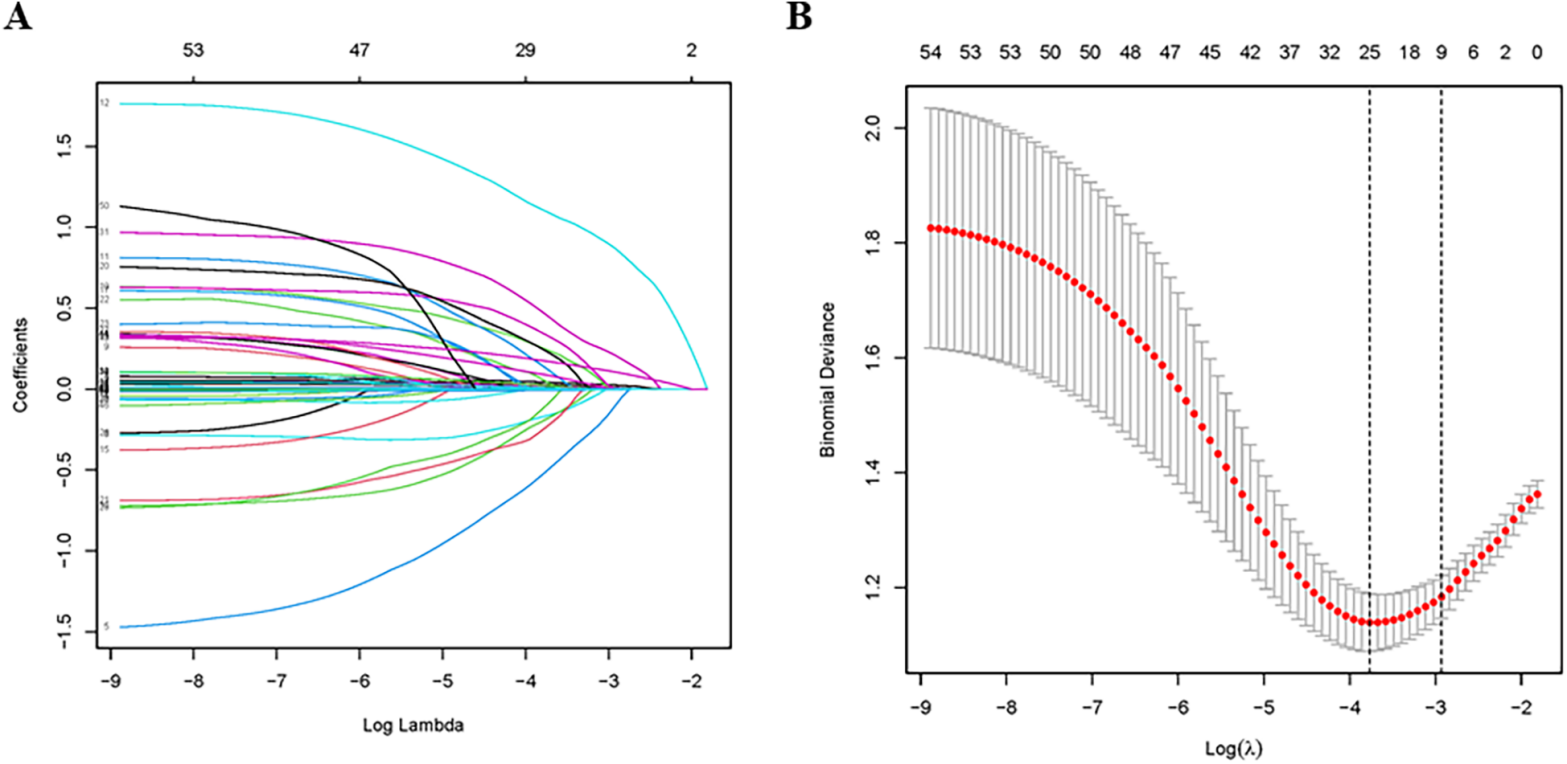
Variable selection using the least absolute shrinkage and selection operator (LASSO) regression model. **(A)** LASSO coefficient profiles of the 54 candidate predictors; **(B)** tuning parameter (*λ*) selection used 10-fold cross-validation in the LASSO model.

The LASSO logistic regression was performed for variable selection and Lambda min was chosen as the best model. 25 variables were selected from 54 variables in the training group (Table 2), including age, witnessed arrest, bystander CPR, time to ROSC, epinephrine, TTM, non-cardiac etiology, non-shockable rhythm, hypertension, COPD/asthma, previous neurological disease, chronic renal failure, liver cirrhosis, CRRT, AKI, lowest ScvO2, lactate, ScvO2/SvO2, alkaline phosphatase, total bilirubin, APTT, proteins, PaO2, MAP and SOFA (Figure 1). We initiated the analysis with a univariate logistic regression on 25 preselected variables. The results of this preliminary analysis retained 12 variables with a significance level of *P* < 0.05 for further examination through multivariate logistic regression, which was conducted following a stepwise regression approach. Ultimately, six independent variables were identified for the construction of the nomogram: age [odds ratio [OR] 1.05, 95% confidence interval [CI] 1.03–1.08], witnessed arrest (OR 0.28, 95% CI: 0.11–0.73), time to return of spontaneous circulation (ROSC) (OR 1.05, 95% CI: 1.02–1.08), non-shockable rhythm (OR 3.41, 95% CI: 1.61–7.18), alkaline phosphatase levels (OR 1.01, 95% CI: 1–1.01), and Sequential Organ Failure Assessment (SOFA) score (OR 1.27, 95% CI: 1.15–1.4). These findings are detailed in Table 3.

**Table 2:** Variables associated with hospital mortality in the training group.

Variables	*N* = 262	Survivor = 108	Death = 154	*P* value
Age, years	62.00 [52.00; 74.75]	59.00 [50.75; 71.00]	65.00 [54.00; 76.00]	0.012
Weight, kg	76.50 [67.25; 85.00]	76.50 [70.00; 85.00]	76.00 [65.00; 85.00]	0.564
Gender, *n* (%)				0.435
Female	71 (27.10%)	26 (24.07%)	45 (29.22%)	
Male	191 (72.90%)	82 (75.93%)	109 (70.78%)	
Hypertension, *n* (%)				0.385
No	143 (54.58%)	55 (50.93%)	88 (57.14%)	
Yes	119 (45.42%)	53 (49.07%)	66 (42.86%)	
Diabetes, *n* (%)				0.435
No	191 (72.90%)	82 (75.93%)	109 (70.78%)	
Yes	71 (27.10%)	26 (24.07%)	45 (29.22%)	
Coronary artery disease, *n* (%)				0.877
No	155 (59.16%)	65 (60.19%)	90 (58.44%)	
Yes	107 (40.84%)	43 (39.81%)	64 (41.56%)	
COPD/Asthma, *n* (%)				0.058
No	218 (83.21%)	96 (88.89%)	122 (79.22%)	
Yes	44 (16.79%)	12 (11.11%)	32 (20.78%)	
Chronic heart failure, *n* (%)				0.958
No	207 (79.01%)	86 (79.63%)	121 (78.57%)	
Yes	55 (20.99%)	22 (20.37%)	33 (21.43%)	
Previous neurological disease, *n* (%)				0.100
No	220 (83.97%)	96 (88.89%)	124 (80.52%)	
Yes	42 (16.03%)	12 (11.11%)	30 (19.48%)	
Chronic renal failure, *n* (%)				1.000
No	222 (84.73%)	92 (85.19%)	130 (84.42%)	
Yes	40 (15.27%)	16 (14.81%)	24 (15.58%)	
Liver cirrhosis, *n* (%)				0.098
No	249 (95.04%)	106 (98.15%)	143 (92.86%)	
Yes	13 (4.96%)	2 (1.85%)	11 (7.14%)	
Corticoids, *n* (%)				0.168
No	201 (76.72%)	88 (81.48%)	113 (73.38%)	
Yes	61 (23.28%)	20 (18.52%)	41 (26.62%)	
Chronic anticoagulation, *n* (%)				0.451
No	219 (83.59%)	93 (86.11%)	126 (81.82%)	
Yes	43 (16.41%)	15 (13.89%)	28 (18.18%)	
Witnessed arrest, *n* (%)				0.049
No	39 (14.89%)	10 (9.26%)	29 (18.83%)	
Yes	223 (85.11%)	98 (90.74%)	125 (81.17%)	
Bystander CPR, *n* (%)				0.153
No	87 (33.21%)	30 (27.78%)	57 (37.01%)	
Yes	175 (66.79%)	78 (72.22%)	97 (62.99%)	
Time to ROSC, min	15.00 [7.00; 25.00]	12.50 [5.75; 20.00]	18.00 [10.00; 26.75]	0.005
Non-cardiac etiology, *n* (%)				0.008
No	153 (58.40%)	74 (68.52%)	79 (51.30%)	
Yes	109 (41.60%)	34 (31.48%)	75 (48.70%)	
Non-shockable rhythm, *n* (%)				<0.001
No	100 (38.17%)	62 (57.41%)	38 (24.68%)	
Yes	162 (61.83%)	46 (42.59%)	116 (75.32%)	
Epinephrine, mg	3.00 [1.25; 5.00]	2.00 [1.00; 4.00]	4.00 [2.00; 7.00]	0.001
Out of Hospital, *n* (%)				0.682
No	107 (40.84%)	42 (38.89%)	65 (42.21%)	
Yes	155 (59.16%)	66 (61.11%)	89 (57.79%)	
TTM, *n* (%)				0.217
no	30 (11.45%)	16 (14.81%)	14 (9.09%)	
yes	232 (88.55%)	92 (85.19%)	140 (90.91%)	
IABP, *n* (%)				1.000
No	244 (93.13%)	101 (93.52%)	143 (92.86%)	
Yes	18 (6.87%)	7 (6.48%)	11 (7.14%)	
ECMO, *n* (%)				1.000
No	227 (86.64%)	94 (87.04%)	133 (86.36%)	
Yes	35 (13.36%)	14 (12.96%)	21 (13.64%)	
Shock, *n* (%)				0.043
No	120 (45.80%)	58 (53.70%)	62 (40.26%)	
Yes	142 (54.20%)	50 (46.30%)	92 (59.74%)	
ICU vasopressor therapy, *n* (%)				0.003
No	66 (25.19%)	38 (35.19%)	28 (18.18%)	
Yes	196 (74.81%)	70 (64.81%)	126 (81.82%)	
ICU Dobutamine, *n* (%)				0.385
No	119 (45.42%)	53 (49.07%)	66 (42.86%)	
Yes	143 (54.58%)	55 (50.93%)	88 (57.14%)	
CRRT, *n* (%)				0.831
No	218 (83.21%)	91 (84.26%)	127 (82.47%)	
Yes	44 (16.79%)	17 (15.74%)	27 (17.53%)	
Amiodarone, *n* (%)				0.465
No	130 (49.62%)	57 (52.78%)	73 (47.40%)	
Yes	132 (50.38%)	51 (47.22%)	81 (52.60%)	
Beta lactams, *n* (%)				0.630
No	147 (56.11%)	63 (58.33%)	84 (54.55%)	
Yes	115 (43.89%)	45 (41.67%)	70 (45.45%)	
AKI, *n* (%)				0.001
No	106 (40.46%)	57 (52.78%)	49 (31.82%)	
Yes	156 (59.54%)	51 (47.22%)	105 (68.18%)	
Lowest ScvO2, %	62.00 [56.00; 66.47]	60.85 [55.35; 65.85]	63.00 [57.00; 67.00]	0.114
Minimum platelets, /mm^3^	132.50 [72.50; 183.25]	133.00 [92.50; 177.75]	132.00 [66.00; 187.00]	0.605
Lactate, mEq/L	5.15 [4.20; 7.60]	4.60 [3.88; 6.12]	5.60 [4.40; 8.67]	0.001
CRP, mg/dl	40.00 [16.50; 80.00]	32.50 [12.00; 73.25]	43.50 [20.00; 86.25]	0.149
Creatinine, mg/dl	1.20 [0.90; 1.60]	1.10 [0.90; 1.50]	1.20 [0.90; 1.80]	0.215
ScvO2/SvO2, %	68.75 [63.28; 74.30]	67.15 [62.45; 72.65]	69.40 [63.70; 75.40]	0.039
AST, IU/L	100.50 [51.00; 208.25]	85.00 [46.75; 209.25]	108.00 [53.00; 205.50]	0.218
ALT, IU/L	72.00 [33.00; 166.00]	74.00 [33.00; 172.75]	72.00 [33.00; 150.50]	0.935
LDH, IU/L	343.50 [245.25; 486.75]	310.00 [235.50; 459.50]	352.00 [251.00; 507.25]	0.079
Alkaline phosphatase, IU/L	74.50 [59.00; 101.75]	69.00 [58.00; 86.00]	82.50 [59.25; 109.00]	0.005
GGT, IU/L	69.00 [43.00; 101.49]	62.50 [40.75; 101.49]	76.50 [45.25; 101.12]	0.169
Total bilirubin, IU/L	0.51 [0.33; 0.87]	0.48 [0.32; 0.78]	0.54 [0.36; 1.10]	0.042
APTT, sec	33.25 [27.40; 44.72]	31.50 [26.17; 44.28]	34.25 [28.60; 45.65]	0.105
PT, sec	61.28 (23.33)	64.92 (23.74)	58.73 (22.78)	0.036
INR	1.30 [1.12; 1.61]	1.24 [1.08; 1.49]	1.34 [1.20; 1.66]	0.012
Platelets, /mm^3^	201.00 [138.25; 250.75]	204.00 [150.75; 249.25]	191.00 [126.25; 258.25]	0.337
Proteins, mg/dl	5.70 [5.10; 6.50]	5.80 [5.30; 6.53]	5.60 [5.00; 6.50]	0.404
Glucose, mg/dl	206.50 [152.25; 293.75]	195.00 [135.75; 275.50]	210.00 [161.25; 303.75]	0.184
PH	7.29 [7.20; 7.38]	7.30 [7.22; 7.38]	7.29 [7.19; 7.38]	0.452
PaO2, mmHg	119.00 [87.00; 183.00]	132.50 [87.00; 178.50]	112.00 [84.00; 185.50]	0.480
PaCO2, mmHg	37.00 [32.00; 44.00]	39.00 [32.00; 45.00]	36.00 [32.00; 43.00]	0.134
MAP, mmHg	86.00 [75.00; 103.00]	93.00 [81.50; 108.00]	83.00 [72.00; 96.75]	<0.001
APACHE II	24.26 (6.93)	23.18 (6.29)	25.02 (7.27)	0.030
SOFA	11.00 [9.00; 14.00]	10.00 [8.75; 12.00]	13.00 [10.00; 15.00]	<0.001

COPD, chronic obstructive pulmonary disease; ROSC, return of spontaneous circulation; APACHE II, acute physiology and chronic health evaluation score; SOFA, sequential organ failure assessment score; ScvO2/SvO2, central venous oxygen saturation or mixed venous oxygen saturation; AST, aspartate aminotransferase; ALT, alanine aminotransferase; LDH, lactate dehydrogenase; GGT, γ-glutamine transferase; APTT, activated partial thromboplastin time; PT, prothrombin time; INR, international normalized ratio; MAP, mean arterial pressure; CRP, C-reactive protein; TTM, targeted temperature management; IABP, intra-aortic balloon counter pulsation; ECMO, extracorporeal membrane oxygenation; CRRT, mechanical ventilation, continuous renal replacement therapy; AKI, acute kidney injury.

**Table 3:** Univariate and multivariate logistic regression analysis for variables selection associated with hospital mortality.

		Univariate			Multivariate	
	OR	95% CI	*P* value	OR	95% CI	*P* value
Age, years	1.02	1–1.04	0.019	1.05	1.03–1.08	<0.001
Witnessed Arrest	0.44	0.2–0.95	0.036	0.28	0.11–0.73	0.01
Bystander CPR	0.65	0.38–1.12	0.119			
Time to ROSC, min	1.03	1.01–1.05	0.008	1.05	1.02–1.08	<0.001
Epinephrine, mg	1.13	1.04–1.21	0.002			
TTM	1.74	0.81–3.73	0.156			
Non-cardiac etiology	2.07	1.24–3.46	0.006	1.76	0.81–3.79	0.151
Non-shockable rhythm	4.11	2.42–6.98	<0.001	3.41	1.61–7.18	0.001
Hypertension	0.78	0.47–1.28	0.32			
COPD/Asthma	2.1	1.03–4.29	0.042	2.44	1–5.96	0.05
Previous neurological disease	1.94	0.94–3.98	0.072			
Chronic renal failure	1.06	0.53–2.11	0.865			
Liver cirrhosis	4.08	0.89–18.78	0.071			
CRRT	1.14	0.59–2.21	0.703			
AKI	2.39	1.44–3.98	0.001	1.82	0.95–3.51	0.073
Lowest ScvO2	1.02	0.99–1.05	0.169			
Lactate, mEq/L	1.11	1.02–1.21	0.012			
ScvO2/SvO2	1.03	1–1.06	0.037	1.03	0.99–1.07	0.096
Alkaline phosphatase, IU/L	1.01	1–1.01	0.01	1.01	1–1.01	0.021
Total bilirubin, mg/dl	1.34	1–1.78	0.048	1.26	0.95–1.68	0.112
APTT, sec	1	0.99–1.01	0.813			
Proteins, mg/dl	1.01	0.98–1.04	0.394			
PaO2, mmHg	1	1.00–1.00	0.468			
MAP, mmHg	0.98	0.97–0.99	0.001	0.99	0.97–1	0.142
SOFA	1.18	1.1–1.28	<0.001	1.27	1.15–1.4	<0.001

COPD, chronic obstructive pulmonary disease; ROSC, return of spontaneous circulation; SOFA, sequential organ failure assessment score; ScvO2/SvO2, central venous oxygen saturation or mixed venous oxygen saturation; APTT, activated partial thromboplastin time; MAP, mean arterial pressure; TTM, targeted temperature management.

### Construction of a prediction nomogram

**Figure 2:**
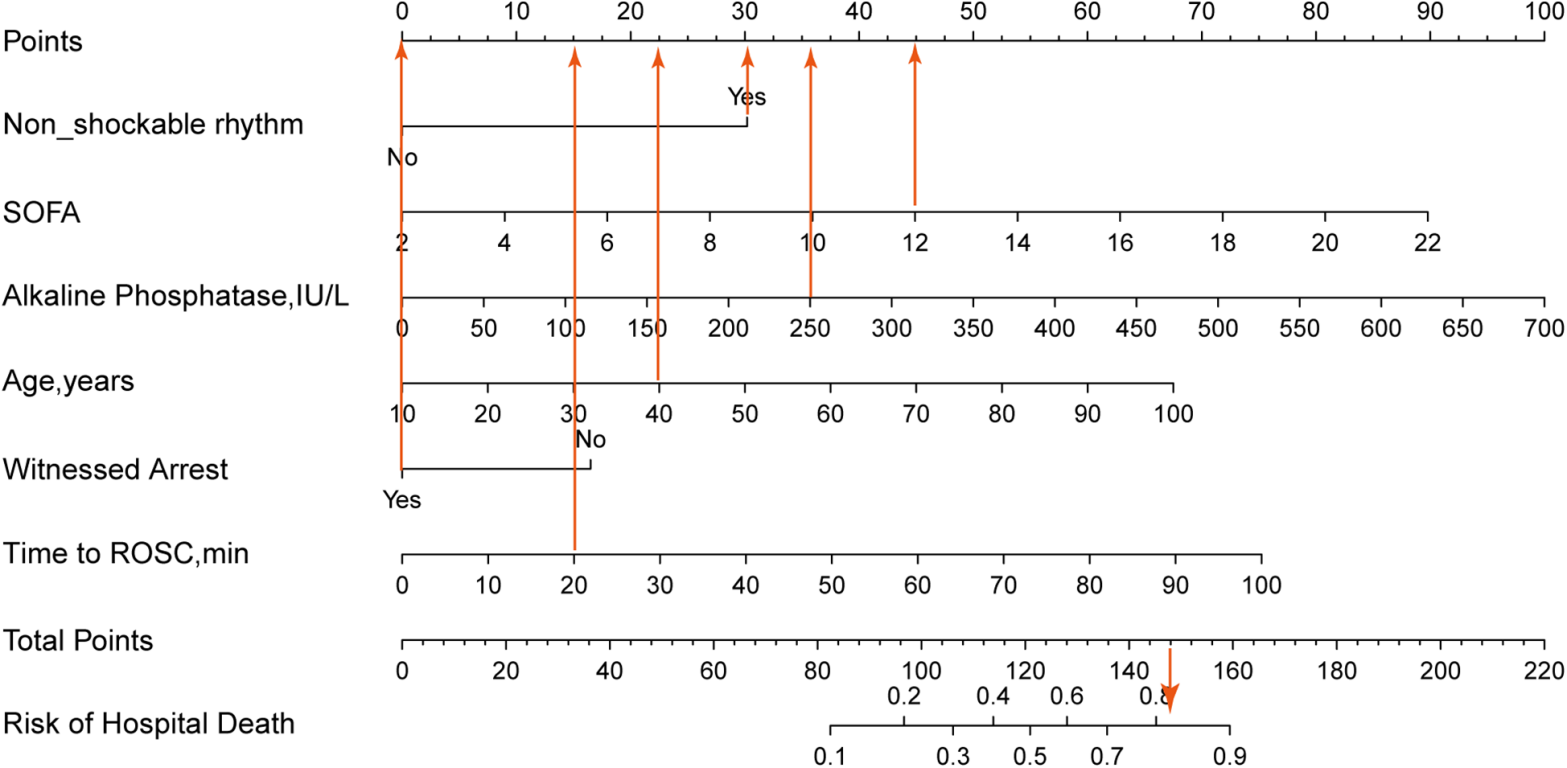
The nomogram was constructed based on the 6 variables from multivariate logistic regression. The nomogram adds up the points for each variable to get a total points and then reflects the probability of death based on the probability of the total points. If a 40-year-old patient had non-shockable rhythm, a SOFA score of 12, alkaline phosphatase of 250 IU/L, with a by-stander witnessed arrest, and with a time of ROSC of 20 min, and the corresponding scores for the various factors would be 22.5, 30, 45, 35.5, 0, and 15, summing up to a total of 148 points. Consequently, this person's risk of hospital mortality is approximately 82%, indicating a relatively high risk of developing hospital mortality.

Based on the 6 independent variables (age, witnessed arrest, time to ROSC, non-shockable rhythm, alkaline phosphatase and SOFA), a prediction nomogram was constructed and we could accurately predict the probability of hospital mortality for individuals with cardiac arrest who survived 24 h after CPR (Figure 2). Each variable was assigned a different score within the range of 0–100 based on its level of importance. We summed the individual scores to calculate a total point. This total point was then converted into a unique risk percentage for hospital mortality, ranging from 0% to 100%. It was proposed that a higher total point from the predictive nomogram signified a greater risk of hospital mortality, whereas a lower total point implied a lesser likelihood of developing hospital mortality. In terms of the clinical application of the nomogram, if a 40-year-old patient had non-shockable rhythm, a SOFA score of 12, alkaline phosphatase of 250 IU/L, with a by-stander witnessed arrest, and with a time of ROSC of 20 min and the corresponding scores for the various factors would be 22.5, 30, 45, 35.5, 0, and 15, summing up to a total of 148 points. Consequently, this person's risk of hospital mortality was approximately 82%, indicating a relatively high risk of developing hospital mortality.

### Evaluation and calibration of the nomogram

**Figure 3:**
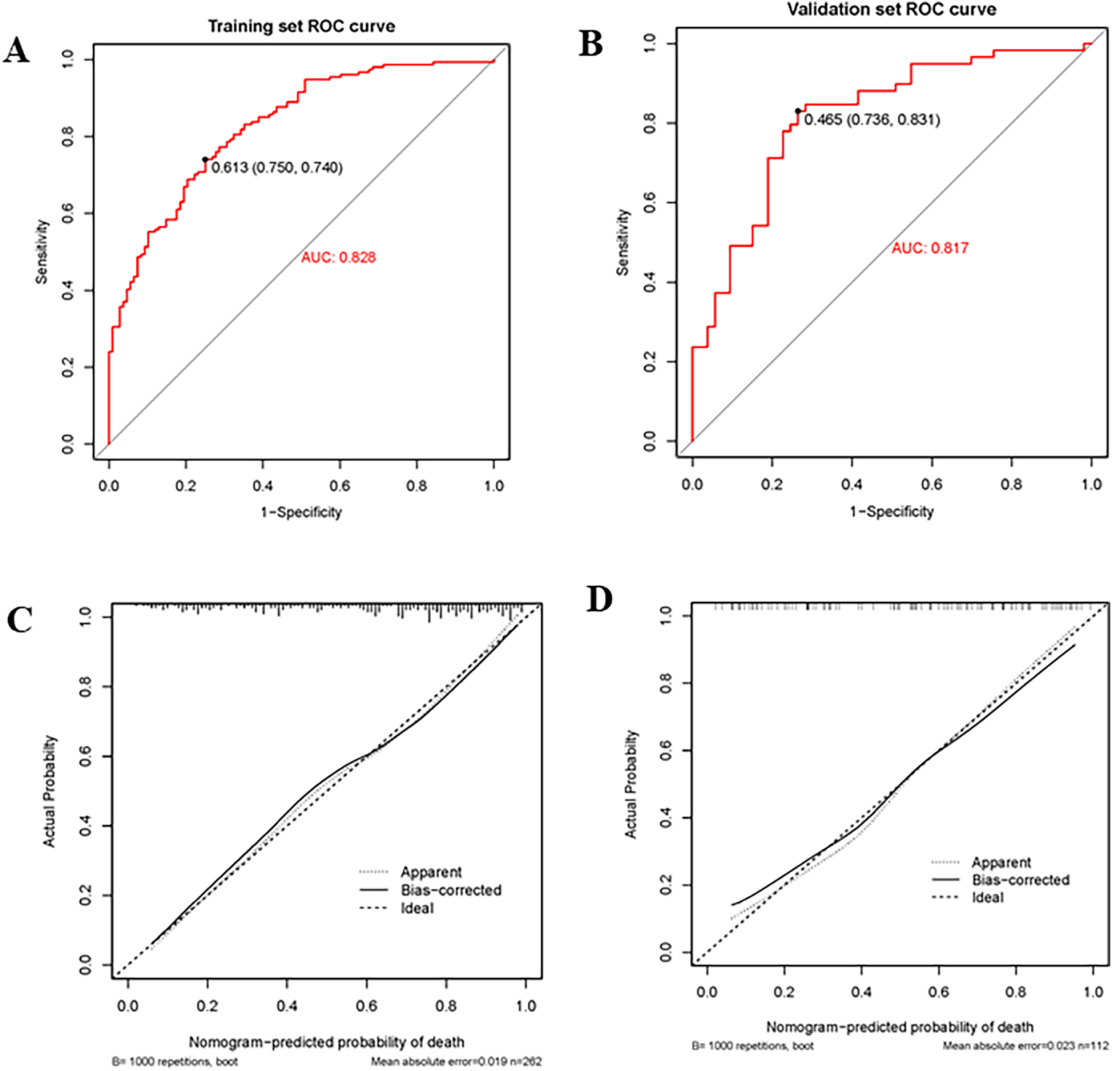
Receiver operating curve (ROC), area under the curve (AUC), and calibration curve in the training group and validation group. **(A,B)** Showed the receiver operating curve (ROC) and area under the curve (AUC) in the training group and validation group; **(C,D)** Showed calibration curves in the training group and validation group. The dashed line was the reference line of the ideal nomogram, the dotted line reflected the performance of the nomogram, while the solid line corrected for any bias in the nomogram.

**Figure 4:**
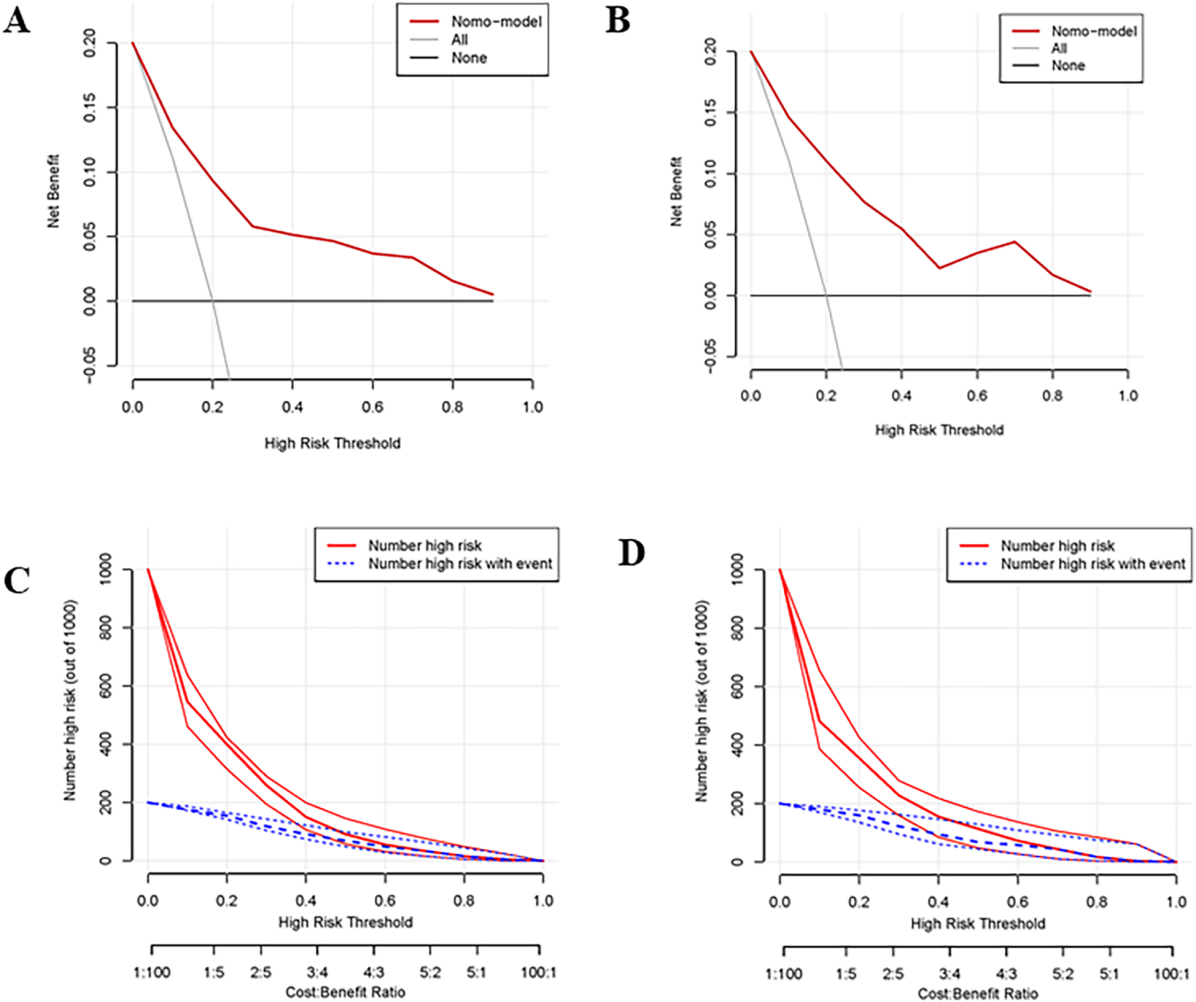
Decision curve and clinical impact curve of the training group and validation group. **(A,B)** Showed the decision curve of the training group and validation group. The black horizontal line indicated that all patients are not treated, so there is no net benefit. The gray sloping line indicated that all patients received treatment, corresponding to the net benefit at different thresholds. The red curve was the net benefit at different risk thresholds based on the risk probability estimated by the constructed model. It can be seen that the net benefit of the model was higher than the net benefit of the horizontal and sloping line within a large threshold range indicating that the prediction value of the model was good. **(C,D)** Showed the clinical impact curve of the training group and validation group. The red curve indicated the number of patients classified as positive (high risk) by the model at each threshold probability, and the blue curve was the number of high risks with an event.

The nomogram had a good discriminating ability in the training group (AUC = 0.827; 95% CI: 0.778–0.876) and validation group (AUC = 0.817; 95% CI: 0.738–0.896) according to the area under receiver operating curves (Figures 3A,B). On the calibration curve, the death probability predicted by the model was close to the observed death probability (Figures 3C,D). The decision curve (Figures 4A,B) and clinical impact curve (Figures 4C,D) in the training and validation group demonstrated that this nomogram had good clinical utility.

## Discussion

We included 374 patients who underwent CPR after cardiac arrest from the database, with 262 patients in the training group and 112 patients in the validation group. Our study indicated that age (OR 1.05, 95% CI: 1.03–1.08), witnessed arrest (OR 0.28, 95% CI: 0.11–0.73), time to ROSC (OR 1.05, 95% CI: 1.02–1.08), non-shockable rhythm (OR 3.41, 95% CI: 1.61–7.18), alkaline phosphatase (OR 1.01, 95% CI: 1–1.01) and SOFA (OR 1.27, 95% CI: 1.15–1.4) were independent critical factors for predicting the risk of death 24 h after CPR. Furthermore, we constructed a nomogram based on these selected factors, which demonstrated good discrimination, accuracy, and clinical utility.

In the early stages of cardiac arrest, multiple organs in the body were still in a better state of oxygenation, and shockable rhythms were more likely to be successfully resuscitated at this stage, thereby increasing the proportion of good prognosis. In contrast, non-shockable rhythms often indicated cardiac energy depletion, failure of function, and a low percentage of successful resuscitation (14, 15). Shockable rhythms, including ventricular fibrillation (VF) and pulseless ventricular tachycardia (PVT), were traditionally regarded as arrhythmias that could be reversed. By administering defibrillation, these rhythms can be corrected to restore the heart's normal rhythm and ensure proper blood flow to organs, thus enhancing the likelihood of survival (14, 16). One study showed that despite the presence of refractory out-of-hospital cardiac arrest, patients with shockable rhythms were more likely to have a lower risk of poor prognosis (180-day mortality), with a hazard ratio (HR) of 0.27 and a 95% confidence interval (CI) of 0.18–0.41 (17). Another study showed that patients with shockable rhythms after sudden cardiac arrest had a higher rate of survival discharged from the hospital (OR 5.21, 95% CI: 2.99–9.07) than those with non-shockable rhythms, after adjusting for sex, age, comorbidities, and ethnicity (18). Non-shockable rhythm typically referred to asystole or pulseless electrical activity (PEA), leading to prolonged ischemia of the brain and other organs, resulting in poor neurological outcomes (19–22).

SOFA score was a simple method for assessing and monitoring organ dysfunction in critically ill patients and had been widely used to assess the severity of critically ill patients and predict their prognosis. The SOFA score comprised six criteria to reflect organ system function (respiratory system, blood system, liver system, cardiovascular system, nervous system, and kidney system) (23). Matsuda et al. (24) conducted a study in 231 cardiovascular arrest patients and found that the SOFA score was lower in those people who survived and SOFA score on admission was a strong predictor of survival at 30 days (OR 0.68, 95% CI: 0.59–0.78). Choi et al. (25) studied the prognosis of 173 patients without-hospital cardiac arrest and found the area under the curve of SOFA score for prediction of 30-day mortality of post-cardiac arrest patients was 0.641 (95% CI: 0.564–0.712). These studies indicated that a decrease in the SOFA score was associated with an increased rate of patient survival, and the SOFA score on admission was significant for predicting the survival rate within 30 days. These findings and our results emphasize the importance of considering the SOFA score in the initial assessment and treatment of patients with cardiac arrest.

When cardiac arrest strikes, time is crucial, with every passing second holding immense significance. Within a span of 4 to 6 min, most patients will start experiencing irreversible brain damage, leading to biological death within a few minutes thereafter. A prompt recognition of cardiac arrest by a bystander, followed by the immediate administration of high-quality CPR, is pivotal in achieving a successful resuscitation outcome. For every 1-minute delay in CPR in cardiac arrest patients, their survival rate decreased by about 10% (26–28). Although numerous studies had demonstrated the importance of bystander CPR when someone witnesses an arrest, the rate of bystander CPR remained relatively low in many countries (29). Nevertheless, we know little about bystanders' perceptions that influenced the decision to start CPR. Our study found that witnessed arrest was independently associated with hospital mortality rate 24 h after CPR, which further reinforced the importance of teaching the early recognition of cardiac arrest.

ROSC was not only a sign of successful CPR but also was the starting point for further treatment and prognosis assessment. In the clinical practice, physicians closely monitored the status of ROSC and adjusted treatment plans accordingly, to improve patient survival rate and quality of life. However, the time to ROSC after CPR varied in different situations, and the ultimate outcomes were also different. In a Swedish cohort study, the median duration of CPR with ROSC was 5 (IQR 2–12) minutes (30). Data from the GWTG-R (Get With the Guidelines-Resuscitation) registry have shown that the median time to achieve ROSC was 11 (IQR 6–21) minutes (31). Although longer duration of resuscitation was associated with worse outcomes, individuals could still benefit from prolonged CPR. Data from the American Heart Association GWTG-R registry showed that 88% of patients achieved sustained ROSC within 30 min (32). These studies indicated that the duration of CPR was closely associated with the success rate of ROSC, but the specific median time to ROSC varied. Therefore, understanding the median time to ROSC can help assess the effectiveness of CPR and may guide clinical decision-making. Our results showed a similar conclusion. The duration from cardiac arrest to the ROSC significantly impacted the survival probability and the shorter duration correlated with improved survival prospects.

Elderly patients were prone to many comorbidities and were therefore more likely to have a poor prognosis after cardiac arrest (33, 34). and some studies had revealed that advanced age was a strong predictor of mortality (31, 35). Sender et al. (36) conducted a study on patients with cardiac arrest in 6 interventional cardiology centers and found that elderly patients over 75 years old were more likely to have adverse neurological function prognosis at 6 months follow-up. Ester et al. (37) retrospectively analyzed 1,285 adult patients with cardiac arrest and found that older patients had worse neurological outcomes and a higher proportion of death after cardiac arrest compared with younger patients. Li et al. (38) retrospectively analyzed the risk factors, and in-hospital outcomes of 320 patients with acute coronary syndrome suffering cardiac arrest and they revealed that age less than 70 was demonstrated to be a strong predictor of survival. However, some studies have found that age was not a risk factor for survival after cardiac arrest, suggesting that when assessing the prognosis of patients who had experienced cardiac arrest, other clinical factors should also be considered in addition to age (39–43).

Alkaline phosphatase (ALP) is a metalloenzyme in membrane-bound glycoprotein, and also an enzyme that catalyzes calcification inhibitor pyrophosphate hydrolysis. ALP is widely distributed in various organs and tissues of the human body, with the highest activity in the liver. The abnormalities can be seen in liver cancer, obstructive jaundice, and other diseases of the liver and bile system, as well as intestinal diseases, metabolic abnormalities, chronic renal insufficiency, and bone metabolism abnormalities (44, 45). Studies have shown that ALP was a new inflammatory mediator of cardiovascular diseases, and the increase of serum ALP level was associated with a variety of atherosclerotic diseases, indicating that ALP was closely related to cardiovascular and cerebrovascular diseases, and its specific mechanism might be leading to increased vascular calcification and vascular dysfunction and thus to promote the release of inflammatory mediators (46–48). Zhong et al. (49) showed that the risk of the early death of stroke patients with high ALP levels was 2.19 times higher than that of stroke patients with low ALP level. Moreover, there was a significant linear relationship between serum ALP level and hospital mortality of cerebral infarction, which further revealed that serum ALP level was associated with poor recovery of neurological function, increased mortality, and overall poor prognosis of cerebral infarction patients. Another study showed that in patients with coronary heart disease, elevated ALP was an independent risk factor for increased 3-year all-cause mortality (50). Therefore, it was speculated that ALP, as an inflammatory indicator, reflected the occurrence and development of cardiac arrest patients to a certain extent, and could be used to predict the prognosis of cardiac arrest patients.

Our nomogram prediction model, based on these selected predictors, had accurate predictive value for hospital mortality in patients who survived 24 h after CPR, which will be beneficial for assisting in identifying high-risk patients and intervention. Compared with the predictive model from Chen et al. (51), we include more variables, up to 54 variables, and thus the predictive model may be more comprehensive and representative.

## Limitations

Our research had some limitations. Firstly, it's an observational study with a relatively small sample size, and selection bias was inevitable, making it difficult to ensure an even distribution of all variables. Secondly, all participants were from Belgium. Further researches were needed to verify whether these findings were also applicable to different ethnicities and external validation data was required to further clarify the accuracy of this model. Thirdly, we analyzed a mixed population of patients with out-of-hospital cardiac arrest and in-hospital cardiac arrest, but there was a significant difference between the two groups, such as cause, prevention, and treatment, and these conditions should be taken into account.

## Conclusion

Our research showed that non-shockable rhythm, SOFA score, witnessed arrest, time-to-ROSC, age, and alkaline phosphatase were significantly associated with hospital mortality for these patients who survived 24 h after CPR. Our nomogram prediction model had accurate predictive value for hospital mortality in patients who survived 24 h after CPR, which will be beneficial for assisting in identifying high-risk patients and intervention. Further confirmation of the model's accuracy required external validation data.

## Data Availability

Publicly available datasets were analyzed in this study. This data can be found here: Dryad Digital Repository (https://datadryad.org/stash/dataset/doi:10.5061/dryad.qv6fp83).
